# A rhamnose-rich O-antigen of *Paraburkholderia phymatum* MP20 is required for symbiosis with *Mimosa pudica*

**DOI:** 10.1128/jb.00422-24

**Published:** 2025-01-23

**Authors:** Shashini U. Welmillage, Euan K. James, Nisha Tak, Sonali Shedge, Lei Huang, Artur Muszyński, Parastoo Azadi, Prasad Gyaneshwar

**Affiliations:** 1 Department of Biological Sciences, University of Wisconsin-Milwaukee 118734 , Milwaukee, Wisconsin, USA; 2 The James Hutton Institute 15554 , Dundee, Scotland, United Kingdom; 3 Department of Botany, Jai Narain Vyas University 563975 , Jodhpur, Rajasthan, India; 4 Complex Carbohydrate Research Center, University of Georgia 123423 , Athens, Georgia, USA; Philipps-Universitat Marburg Fachbereich Biologie, Marburg, Germany

**Keywords:** lipopolysaccharide, nodulation, nitrogen fixation, plant growth promoting rhizobacteria (PGPR), GUS, GFP

## Abstract

*Paraburkholderia phymatum,* a β-proteobacterium, forms a nitrogen-fixing symbiosis with many species of the large legume genus *Mimosa* as well as with common bean (*Phaseolus vulgaris* L.). *Paraburkholderia* are considered to have evolved nodulation independently from the well-studied α-proteobacteria symbionts of legumes. However, the detailed mechanisms important for β-rhizobia-legume symbiosis have not yet been determined. In this manuscript, we have sequenced the genome of *P. phymatum* MP20, a strain isolated from *Mimosa pudica* nodules, and utilized transposon mutagenesis to identify a mutant that showed delayed and ineffective nodulation of *M. pudica*. Further analysis revealed that the mutant strain produced an altered lipopolysaccharide lacking rhamnose containing O-antigen. Complementation with the wild-type gene restored the symbiosis. Microscopic analysis of the ineffective nodules showed that the mutant strain did not infect the cortical cells but was restricted to the endodermis. The results suggest that the O-antigen of *P. phymatum* is important for the bacterial infection of cortical cells and for nodule maturation. Further research will unveil the specific involvement of the glycosyltransferase gene in LPS biosynthesis and its impact on successful nodule formation by *P. phymatum*.

**IMPORTANCE:**

The nitrogen-fixing symbiosis between legumes and rhizobia is important for agricultural and environmental sustainability. The mechanisms of the symbiotic interactions are extensively studied using α-rhizobia. In contrast, mechanisms of symbiotic interactions important for β-rhizobia and their Caesalpinioid (mimosoid) legume hosts are not well known. Here, we describe the genome sequence of *P. phymatum* MP20*,* a β-rhizobia isolated from the nodules of *M. pudica,* and isolation and characterization of a transposon mutant defective in symbiosis. We demonstrate that the O-antigen of the LPS is required for nodulation and symbiotic nitrogen fixation. This study broadens our knowledge of symbiotic interactions in β-rhizobia and will lead to a better understanding of the wider rhizobial-legume symbiosis apart from the α-rhizobia.

## INTRODUCTION

Plants belonging to the Leguminosae (Fabaceae) family are one of the major contributors to the global N economy through their ability to perform symbiotic biological N_2_ fixation (BNF) in agricultural and natural environments. Legumes are important food and feed crops. They include soybeans (*Glycine max*), beans (*Phaseolus vulgaris*), peas (*Pisum staivum*), alfalfa (*Medicago sativa*), and many others. These plants form symbiotic associations with soil bacteria, collectively known as rhizobia, resulting in the formation of specialized structures called nodules in which the rhizobia fix N_2_ for the plant in exchange offor carbon (1–3). Initiation of this symbiosis requires signal exchanges between the host plant and its microbial partner. Legume roots secrete (iso) flavonoids that activate the expression of bacterial *nod* genes (4, 5). *nod* gene activity leads to the production of rhizobial signal molecules, called Nod factors, that are recognized by the legume, leading to root hair deformations, nuclear calcium spiking, and “nodulin” gene expression (4–8).

Until recently, rhizobia were considered to belong to a limited number of genera within four families belonging to α-proteobacteria (α- rhizobia) (9, 10). However, since 2001, a considerable body of evidence has accumulated to show that legumes, particularly those in the genus *Mimosa*, form effective nodules with the distantly related β-Proteobacteria (β-rhizobia) belonging to *Cupriavidus* and *Paraburkholderia* (3, 11–14). In addition to *Mimosa* and other mimosoids (15), β-rhizobia are also symbionts of some papilionoid legumes such as *Cyclopia* and its relatives as well as some members of tribes *Phaseoleae, Crotalarieae* and *Indigoferae* (16–20), and *Rhynchosia* (17). The crop legume *P. vulgaris* (common bean) can also form symbioses with *Paraburkholderia* (21, 22).

The current understanding of the bacterial mechanisms important for nodulation and symbiotic nitrogen fixation is based upon α-rhizobia and their papilionoid legume hosts. In contrast, not much is known about the mechanisms that β-rhizobia utilize for symbiosis with their mimosoid legume hosts. *P. phymatum* is being used as a model to determine nodulation mechanisms of β-rhizobia as it is a highly competitive symbiont of *Mimosa pudica* and other *Mimosa* spp. (13, 14, 23). *M. pudica* is a highly invasive weed throughout the pantropics and the rhizobial symbiosis assists in its invasiveness (3, 24, 25). Therefore, knowledge about its symbiosis is potentially important for potential bio-control.

The genome of *P. phymatum* STM815^T^ consists of two circular chromosomes and two plasmids, one containing the genes important for symbiosis (26). Recent studies have shown that *P. phymatum* nodulation of *M. pudica* is independent of the quorum sensing Bra I/R system (27), and the Typ6 secretion system is not essential for nodulating *Vigna unguiculata* (28). However, nitrogen starvation-specific sigma factor is required for symbiosis with beans (29). We have earlier isolated *P. phymatum* MP20 (hereafter called MP20) from nodules of *M. pudica* growing in India and described its symbiotic properties (24). We are utilizing MP20 to determine the mechanisms important for symbiotic interactions as it is a natural isolate of *M. pudica* nodules as compared to *P. phymatum* STM815^T,^ which was isolated from *Machaerium lunat*um. In this study, we used pMiniHimar1 transposon to generate insertion mutants of MP20 marked with GUS (24) and screened for defects in nodulation and plant growth promotion. We characterize a mutant strain that showed delayed nodulation and ineffective symbiosis and demonstrate that rhamnose containing O-antigen of lipopolysaccharide (LPS) is important for symbiotic compatibility of *P. phymatum* and *M. pudica*. This is the first study to report the composition of LPS from *Paraburkholderia* spp. and its role in nodulation and symbiosis.

## MATERIALS AND METHODS

### Bacterial strains and growth conditions

The bacteria strains, plasmids, and primers used in this study are listed in Table 1. *P. phymatum* strains were maintained in Yeast Extract Mannitol (YEM) or Tryptone Yeast Extract (TY) media. The *E. coli* strains were maintained in Luria agar. The rhizobia were grown in TY broth for plant inoculations, washed, and resuspended in sterile deionized distilled water. The plasmid containing red fluorescent protein was transferred to Tn51 using triparental mating as described earlier (30). Antibiotics were added at the following concentrations (μg mL^−1^). Ampicillin (100), Kanamycin (25), and Tetracycline (10) were used for *E. coli,* and Kanamycin (100) and Tetracycline (5) were used for MP20.

**TABLE 1:** Strains, plasmids, and primers used in this study

Strains	Description
MP20-GUS	*phymatum* MP20 pCAM121::gusA Spec^r^
MP20-GUS-GFP	MP20-GUS with pHC60 Spec^r^ Tet^r^
TN51	MP20 (GUS) *Himar* mutant; Kan^r^
TN51-RFP	TN51 marked with pBHR-RFP; Kan^r^ Tet^r^
*E. coli* DH5α	*E. coli* strain for plasmid construction
*E. coli* H101 pRK2013	Helper strain for triparental mating
*E. coli* DH5α pBHR	*E. coli* with pBHR mRFP. Tet^r^
Plasmids	
pMiniHimar	*mini-HimarRB1* transposon, R6K *ori* Kan^r^
pGEM-T Easy	Cloning vector; Amp^r^
pBBR1MSC-3	Broad-host-range vector; Tc^r^
pBluescript-SK	Cloning vector; Amp^r^
pBBRIMSC-3::TN51 operon	pBBRIMSC-3 with the wild-type copy of the putative operon where transposon got inserted to complement the mutation
pBHR mRFP	Constitutively expressed RFP, Tet^r^
Primers	
Himar1	CATTTTAATACTAGCGACGCCATCT
Himar 615	TCGGGTATCGCTCTTGAA
k2	CGGTGCCCTGAATGAACT
Kt	GGGCCACAGTCGATGAATCC
Tn51gst_F	AGCAGCTCGCACAGACACTG
Tn51gst_R	GAAGAACCGGAACGCCGAC
Tn51operon_FSpeI	GCGACTAGTAACGCTCTGCCAGTTTCTC
Tn51operon_RSacI	CGAGCTCCTTCTGACACCAGGTAAG
Gtf2_RTF	GCTACTATCTTGTGCTGAATCCG
Gtf2_RTR	GCAGAGGTACTGGATGCTGC
Poly_RTF	CTCCGAGTACGCGATGTACC
Poly_RTR	CTCCGAGTACGCGATGTACC

### Plant germination and growth conditions

The *M. pudica* seeds (obtained from Outsidepride Seed Source, USA) were sterilized with concentrated sulfuric acid for 5 min, washed five times with sterile water, and germinated on sterile paper towels at 30°C for 48 h. The germinated *M. pudica* seedlings were grown individually in 10 mL glass tubes containing N-free plant growth media (modified Jensen’s N-free medium). The plants were incubated in a growth chamber at 26°C with a 14/10 h light/dark cycle.

### Plant nodulation assay

*Mimosa pudica* seeds were germinated as described before, and the seedlings were planted in Styrofoam cups containing sterile vermiculite, one seedling per cup. Two days after transplanting, five replicates were inoculated with 1 mL of a cell suspension of 1.0 OD_600_. Uninoculated plants served as control. Nodulation was observed 7, 14, and 28 days after inoculation (dai).

### Genome sequencing and phylogenetic analysis of *P. phymatum* MP20

The sequencing of the MP20 genome was performed at MicrobesNG (http://www.microbesng.com) using Illumina HiSeq 2500. The genome was sequenced using 2 × 250 bp paired-end reads (30× coverage). The quality control and *de novo* assembly were performed per company protocols. The genome sequence was deposited in GenBank (accession number: JBDXTG000000000). The average nucleotide identity (ANI) of strain MP20 with selected *Paraburkholderia* spp. was calculated using ANI/AAI-Matrix (31) and the OrthoANIu algorithm (32). A phylogram was prepared based on ANI-Distance clustering using the BIONJ method. The probability of two genomes belonging to the same species was done using JSpeciesWS (32). The FastANI method was also used, which estimates ANI in the 80%–100% identity using alignment-free approximate sequence mapping (33). Type (Strain) Genome Server (TYGS) was used to analyze genome-based taxonomy (34) and to calculate the pairwise digital DNA-DNA hybridization (dDDH) values between the genome of MP20 and the selected genomes (35). For genome sequence-based species delimitation, the dDDH similarity values were calculated using three different Genome BLAST Distance Phylogeny (GBDP) formulas (36).

### Construction and screening of MP20 transposon mutant library

The mutant pool was generated using pMiniHimar transposon as described earlier (37). The transposon was moved into MP20-GUS using biparental conjugation, and the transconjugants were selected on a YM minimal medium containing kanamycin. Seven hundred individual mutant colonies on the selection plates were grown in YM minimal broth with kanamycin in 96-well plates and stored at −80°C. The individual mutants were inoculated onto *M. pudica* seedlings and monitored for nodulation and symbiotic N-fixation, as described earlier (24). A 200 µL cell suspension at an OD_600_ value of 1.0 was inoculated into each glass tube containing one *M. pudica* seedling. The exact same amount of MP20 was inoculated onto the positive control plants. Negative controls were uninoculated. After 14 days, the number of pink (active) nodules was counted for each mutant. Approximately, 300 mutants were screened. A mutant (hereafter known as TN51) showing delayed and inactive nodule formation was identified, named, and further characterized.

### Identification of transposon insertion site

Genomic DNA was extracted from TN51 using the Wizard Genomic DNA Purification Kit according to the manufacturer’s protocol. To identify the transposon insertion site, genomic DNA was digested with the PstI enzyme, which digests at one site within the transposon. The digested DNA was ligated to the PstI digested pBluescript-SK plasmid and was transformed via electroporation into *E. coli* DH5α electrocompetent cells. The transformed cells were selected on LB agar with kanamycin.

### Microscopy

The roots of *M. pudica* seedlings inoculated with MP20-GUS and by the TN51 mutant were collected and stained for GUS activity using X-Gluc (38). The nodules and nodule-like outgrowths that stained blue were cut into small pieces and fixed in 4% glutaraldehyde in 50 mM phosphate buffer (pH 7.0). The fixed samples were prepared for light and transmission electron microscopy (TEM) as described (38, 39). The sections were viewed under a Zeiss Axiophot 2 optical microscope, and the ultrathin sections were viewed using a JEOL JEM 1400 TEM. Fluorescent microscopy was used to visualize the MP20 strain marked with green fluorescent protein (GFP) using an I3 filter (excitation, 450–490 nm and emission at 515 nm) and the Tn51 mutant marked with red fluorescent protein (RFP) using an N2.1 filter (excitation at 515–560 nm and emission at 590 nm). Freehand sections of 8-week-old nodules were prepared and observed under a Leica TCS SP2 (Leica Microsystems, Bannockburn, IL).

### Autoagglutination assay

The MP20, Tn51 mutant, and the Tn51 complemented strain were cultured in Tryptone Yeast broth at 28°C with shaking overnight until approximately OD_600_ = 0.9. A final concentration of 1% NaCl was added to one half of the culture; the other half was left as the negative control. The cultures were then left stationary at room temperature overnight. The top 1 mL was removed without agitation, and the OD_600_ was measured (A*_t_*). The remainder was vortexed vigorously, 1 mL of the cell suspension was removed, and the OD_600_ was measured (A_0_). Percentage autoagglutination was calculated using the following equation:


$$\%\;Autoagglutination=\left(1-\left(\frac{A_{t}}{A_{0}}\right)\right)\times 100.$$

### LPS isolation

LPS extraction and characterization were performed at the Complex Carbohydrate Research Center, University of Georgia. The LPS was extracted from cells using hot phenol, following the Westphal and Jann procedure (40). Briefly, an equal volume of hot phenol (68°C) was added to the cell suspension and extracted for 20 min at 68°C by gentle stirring. The sample was then cooled on an ice bath and centrifuged for 20 min at 4°C, 10,000× *g*. The aqueous upper phase was collected, and the remaining phenol phase was extracted two more times with water. The combined aqueous phases and the phenol phase were dialyzed against several water exchanges for four days (12–14 kDa MWCO) to remove residual phenol and freeze-dried. The crude LPS was resuspended in a sterile buffer (50 mM MgCl_2_•6H_2_O and 20 mM NaOAc•3H_2_O, 0,1% sodium azide). Nucleic acids were digested with Benzonase (125u) for 16 h at 37°C with gentle agitation and proteins with Proteinase K (50 µg/mL—per mL of the LPS solution) for 16 h at 37°C. Finally, the digested samples were dialyzed against several exchanges of water (12–14 kDa MWCO) at 4°C, followed by ultracentrifugation at 100,000× *g* for 18 h at 4°C. The LPS-enriched pellets and the supernatants were freeze-dried.

### DOC-PAGE analysis and chemical characterization of LPS

The purified samples were mixed with Laemmli buffer and 1 µg of each was resolved in PAGE (4% stacking gel and 18% resolving gel) in the presence of the deoxycholic acid buffer (41). The PAGE was visualized with a silver stain reagent kit (Bio-Rad) or with Alcian Blue without periodate oxidation and stained with silver (42, 43). The glycosyl and fatty acid composition was done by the conversion to O-trimethylsilyl (TMS) methyl derivatives and fatty acid methyl esters (FAME and TMS-FAME), after methanolysis with 1 M HCl-methanol at 80°C for 18 h, followed by re-N-acetylation and O-trimethylsilylation (44). Myo-inositol was used as an internal standard. TMS and FAME derivatives were resolved by gas chromatography-mass spectrometry (GC-MS) on an Equity-1 (Supelco) fused silica capillary column (30 m length × 0.25 mm ID × 0.25 µm film thickness) on an Agilent AT 7890A GC system interfaced to a 5975B MSD. The oven temperature was set at 80°C for 2 min, then increased to 140°C at 20 °C/min with 2 min hold, and to 200°C at 2 °C/min, followed by an increase to 250°C at 30 °C/min with 5 min hold.

### Statistical analysis of data

The data were analyzed for statistical significance using ANOVA and *t*-tests.

## RESULTS

### Genome sequencing and species determination for MP20

Strain MP20 was earlier identified as *P. phymatum* based only on 16S rDNA sequence analysis (24). To better establish its identity, we determined the whole genome sequence of MP20 and compared it to genomes of other *Paraburkholderia* spp. In accordance with the 16S rDNA analysis, the phylogenomic tree inferred with FastME from GBDP distances using TYGS demonstrates that MP20 is closest to the type strain of *P. phymatum* STM815^T^ (Fig. S1). In addition, genome comparisons using multiple tools showed that the average nucleotide identity between MP20 and *P. phymatum* STM815^T^ ranged between 99.9% and 100% (Fig. S2).

### Isolation of a mutant showing late nodulation and ineffective nitrogen fixation

To determine genes important for the nodulation of *M. pudica*, random transposon-insertion mutants in the MP20-GUS were generated. To reduce the number of mutants to screen for nodulation defects, the mutants were selected on minimal media to eliminate auxotrophs. 400 individual mutants were inoculated separately onto *M. pudica* seedlings, and their ability to nodulate was determined 10 dai. After multiple screening of mutants showing nodulation defects, one of them (Tn51) that consistently failed to form a symbiotic relationship as determined by delayed nodule formation and inefficient N-fixation (as determined by yellowing of leaves) was selected for further investigation.

**Fig 1:**
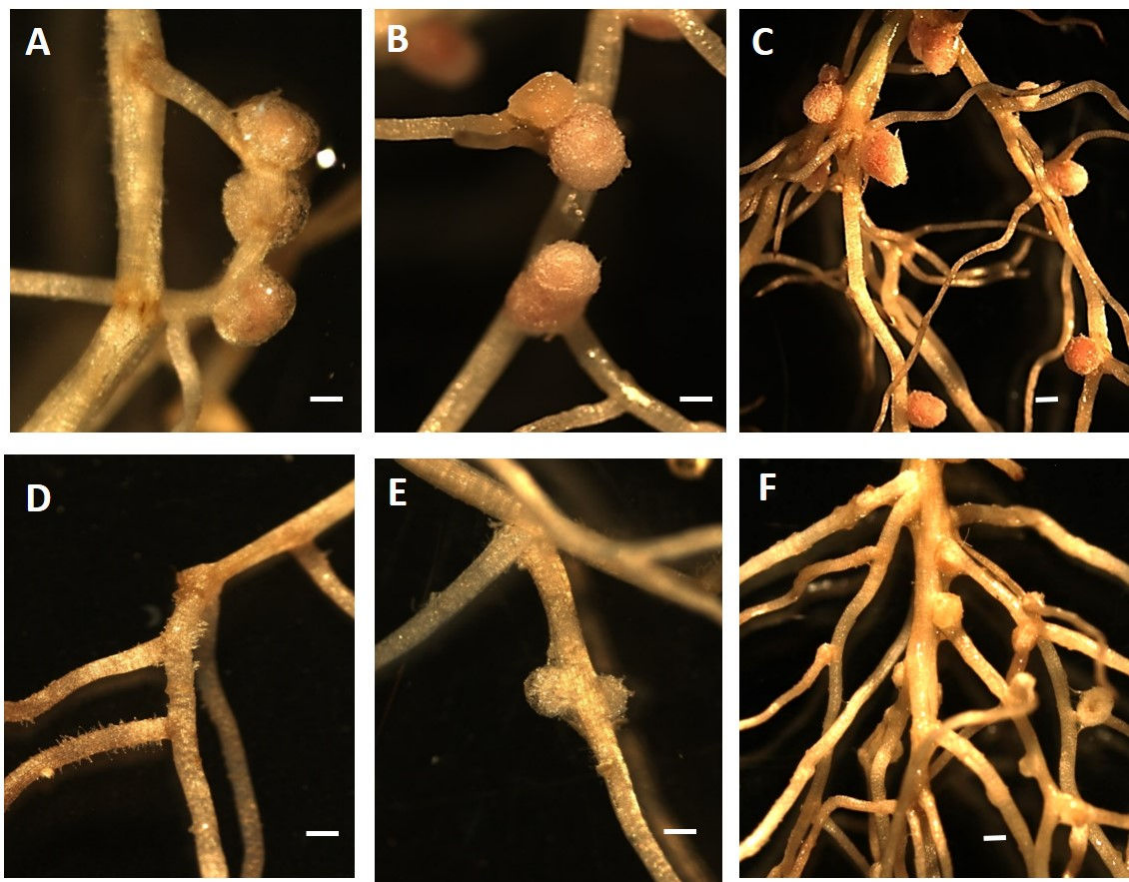
Nodulation of *M. pudica* by *P. phymatum* MP20 and transposon mutant Tn51. *P. phymatum* MP20 formed nodules at 7 dai (**A**) that showed pink coloration (effective for N-fixation) by 14 dai (**B**) and 28 dai (**C**). In contrast, no nodules were observed for the Tn51 mutant at 7 daiI (**D**). Small white nodules were seen at 14 dai (**E**) and 28 dai (F). Bar = 1 mm.

The MP20 and Tn51 strains were inoculated onto *M. pudica* seedlings, and nodulation was evaluated at 7, 14, and 28 dai. Uninoculated plants served as controls. Plants inoculated with MP20 formed nodules by 7 dai, which became symbiotically effective (pink) by 14 dai (Fig. 1A and B). In contrast, no nodules were detected on the plants inoculated with strain TN51 at 7 dai (Fig. 1D), but by 14 dai, small white nodule-like outgrowths could be detected (Fig. 1E), and these remained small and white until 28 dai (Fig. 1F) while nodules on plants inoculated with MP20 were pink and large (Fig. 1C). No nodules formed on the uninoculated controls.

**Fig 2:**
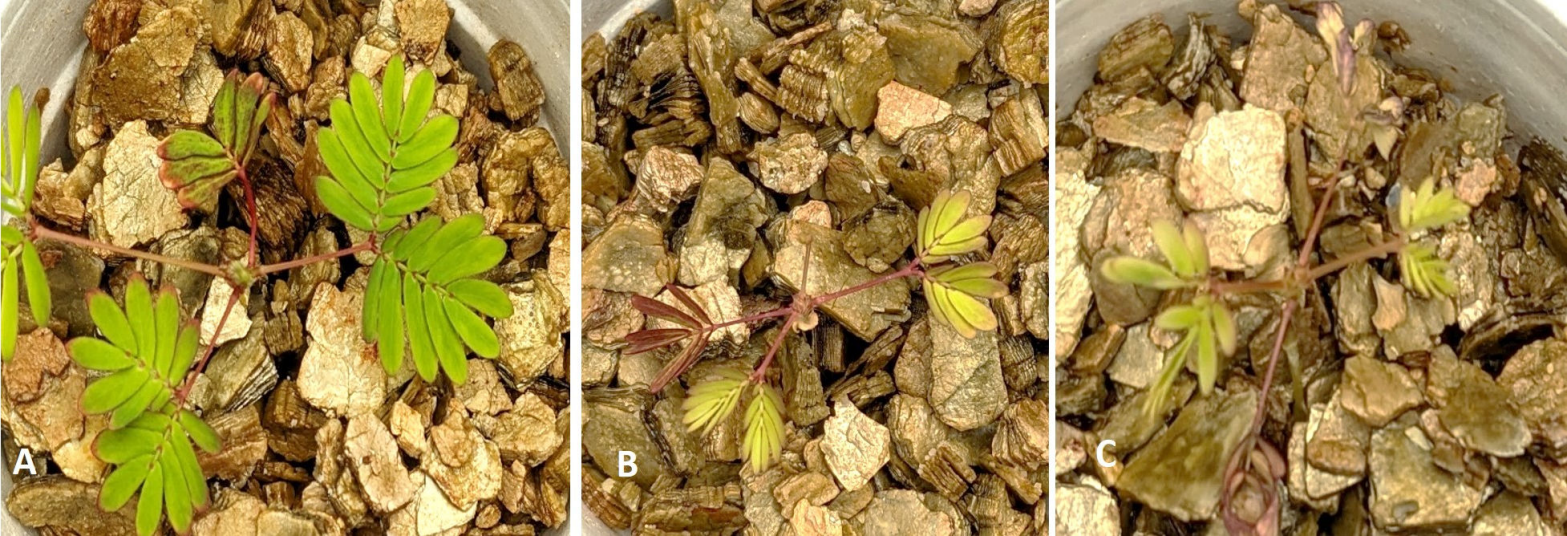
Growth enhancement of *M. pudica* by *P. phymatum* MP20 and transposon mutant Tn51. (A) Plant inoculated with *P. phymatum* MP20. (B) Plant inoculated with Tn51 mutant. (C) Uninoculated control. Note the increased growth and green leaves of plants inoculated with *P. phymatum* MP20 but not plants inoculated with Tn51 mutant.

As the Tn51 strain formed white nodule-like structures, we evaluated its symbiotic effectiveness by analyzing the effect of inoculation on plant growth in the absence of added nitrogen. The leaves of the plants inoculated with the wild-type strain were healthy and green, whereas those of the Tn51-inoculated plants were smaller and yellow (signs of N-deficiency) and very similar to the uninoculated control plants in shoot dry weight (Fig. 2 and 6). These results show that the Tn51 strain was delayed in nodule formation, and the nodules formed were ineffective in enhancing plant growth and most likely did not fix N either.

### Anatomical analysis of *M. pudica* nodulation by the Tn51 and MP20

**Fig 3:**
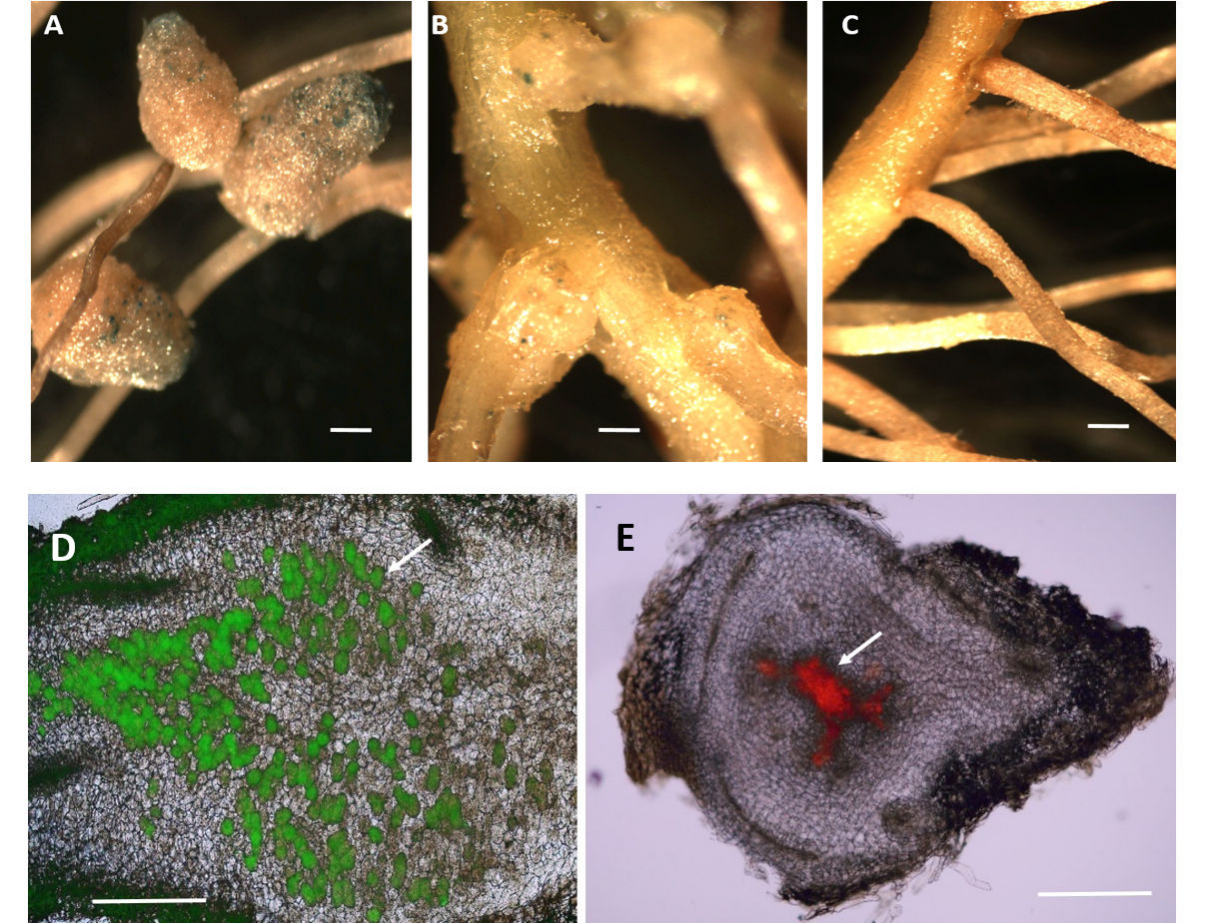
Colonization of *M. pudica* nodules by M20 and Tn51. (A) Nodules formed by *P. phymatum* MP20 showed GUS staining (blue color) throughout the nodule. (B) Nodules formed by the Tn51 mutant have much less staining than the MP20 (compare A and B). (C) N uninoculated roots do not show GUS staining. Bar = 1mm. (D) *P. phymatum* MP20 shows robust colonization of cortical cells as determined by GFP fluorescence (green color). (E) Tn51 mutant colonized only a few cells in the nodules as seen by RFP fluorescence (red color). Bar = 100 µm.

To determine if the GUS-marked bacteria had colonized the small nodules formed by Tn51, the nodules were stained with X-gluc, a GUS substrate. The nodules turned blue, indicating GUS activity of the bacterial symbionts. However, the intensity of the blue color was much less than those formed by the wild-type strain (Fig. 3A and B), indicating that the Tn51 strain was present inside the nodules but possibly at a lower population than the MP20-infected nodules. To further study the colonization, we marked the Tn51 strain with a plasmid containing RFP and compared its nodulation to MP20 marked with GFP using fluorescent microscopy. Nodules were freehand sectioned using a razor blade and observed under the fluorescent microscope. In the nodules formed by the MP20 strain, the GFP-marked bacteria were present in the infected cortical cells (Fig. 3D). In contrast, the nodules induced by TN51 did not form structures typical of a mature indeterminate *Mimosa* nodule, with the RFP-marked bacteria concentrated in the center of the nodule (Fig. 3E).

**Fig 4:**
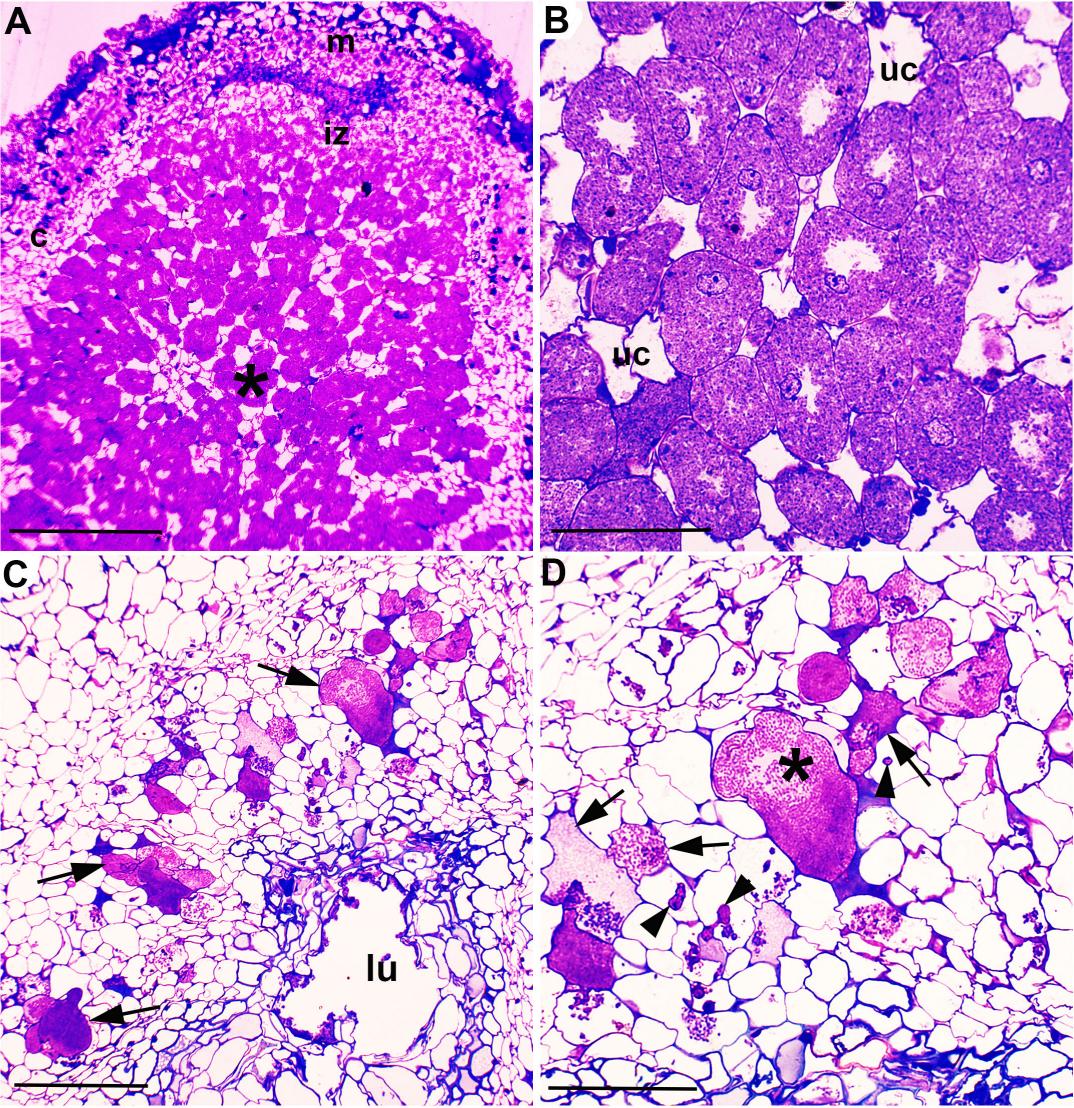
Light microscopy of nodule infection by *P. phymatum* MP20 and Tn51 mutant. (A) and (B) *Mimosa pudica* nodule infected with *P. phymatum* MP20. (C) and (D) Structure formed on a *M. pudica* root after inoculation with the LPS-deficient mutant of *P. phymatum* MP20. (A) Longitudinal profile of a symbiotically effective *M. pudica* nodule indicating the various zones of an indeterminate nodule. m = meristem, iz = invasion zone, and * indicates the N-fixing zone. Bar = 200 µm. (B) Detail of the infected, N-fixing cells; note that these cells are large, packed with bacteroids, and blue-stained, whereas the uninfected (interstitial) cells are smaller, apparently empty, and unstained (uc). Bar = 50 µm. (C) Note that the non-symbiotic structure formed by the LPS-deficient mutant lacks the organized structure of the nodule shown and has signs of degradation, e.g., a large lumen (lu) has formed in its center. Bacteria have infected it and colonized some of the cells (arrows). Bar = 100 µm. (D) A higher magnification view of (C) indicates a large cell apparently packed with bacteria (*), but also collapsed plant cells and/or large conglomerations of intercellular bacteria (arrows). Note the small structures resembling infection threads (arrowheads). Bar = 50 µm.

**Fig 5:**
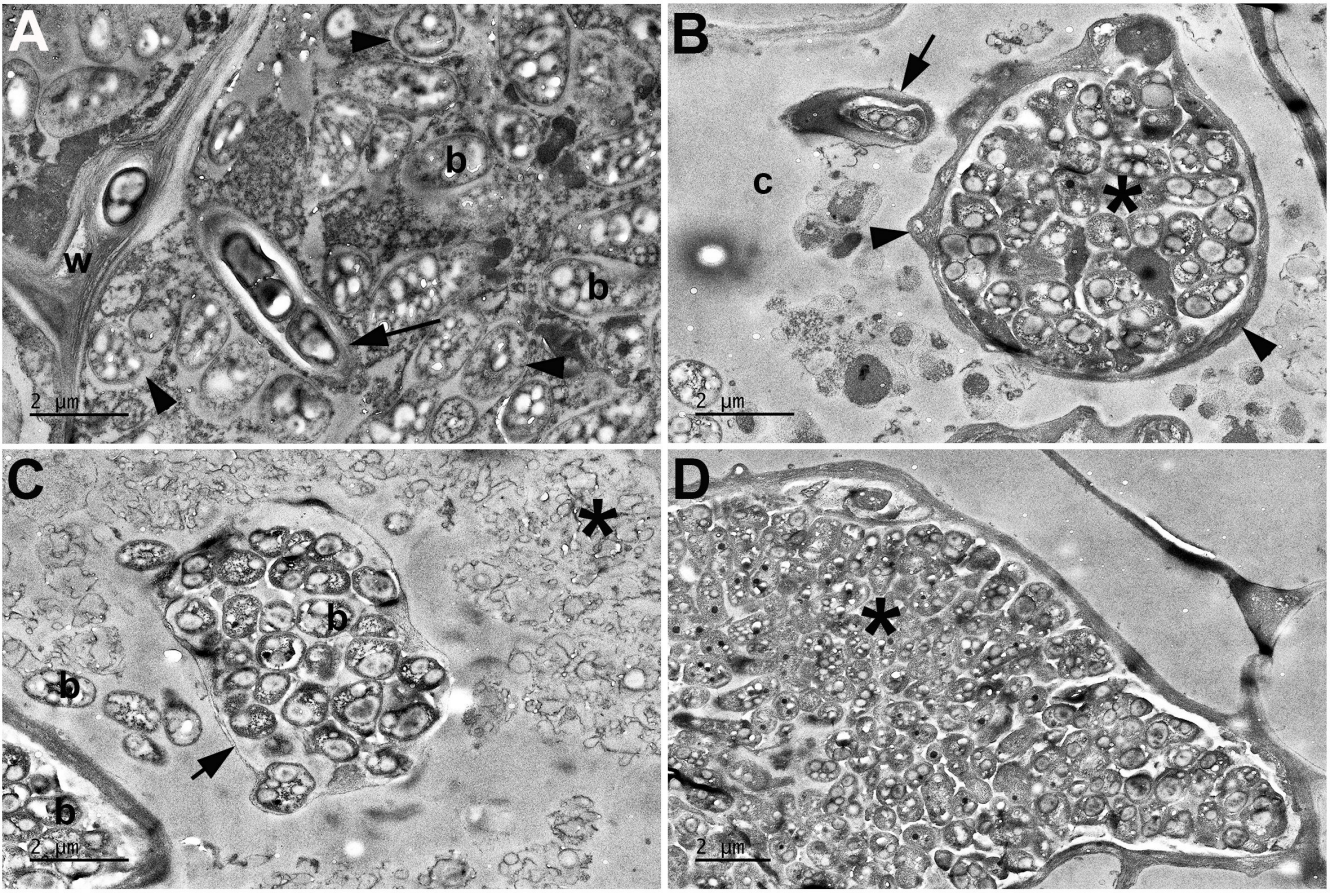
Transmission electron microscopy of nodule infection by *P. phymatum* MP20 and Tn51 mutant. (A) *Mimosa pudica* nodule infected with *P. phymatum* MP20. (B)–(D.) The structure formed on an *M. pudica* root after inoculation with the LPS-deficient mutant of *P. phymatum* MP20. (A) Infected cell in a symbiotically effective *M. pudica* nodule. The bacteria infect the cell in an organized manner via an infection thread (arrow) and then become established as bacteroids (b) enclosed within membrane-bound symbiosomes (arrowheads). W = cell wall. (B) An infection thread-like structure (arrow) is present in a cell of the structure shown in (C and D), as is a large conglomeration of bacteria (*) enclosed within a cell wall (arrowheads). (C) Intact bacteria (b) within a host cell containing the remnants of degraded/lysed bacteria (*). Note that most of the intact bacteria are contained within a structure surrounded by a thin cell wall or a membrane (arrow). (D). Bacteria (*) are packed into an enlarged intercellular space. Bar = 2 µm.

Semi-thin (1 µm thickness) sections of resin-embedded nodules were observed under a light microscope to further investigate the nodule ultrastructure. The nodules of the MP20 strain showed a typical indeterminate nodule structure with cortical cells infected with the bacteroids similar to the nodules formed by *P. phymatum* STM815^T^ and by other beta-rhizobia strains that nodulate *M. pudica*, such as *Cupriavidus taiwanensis* (11) (Fig. 4A and B). In contrast, the nodules formed by Tn51 did not show well-organized zones, the bacteria were restricted to the endodermis, and only a few cortical cells were infected (Fig. 4C and D). Higher resolution analysis under the TEM revealed that the MP20 strain invaded host cells in an organized manner via infection threads and formed bacteroids in membrane-bound symbiosomes (Fig. 5A). In contrast, the Tn51 mutant failed to form symbiosomes, and the infected cells showed signs of degradation, indicating a pathogenic response (Fig. 5B through D).

The anatomical observations indicated that the Tn51 mutant is likely affected in its ability to survive within nodules. To determine the viability of bacteria inside the nodules, nodules formed by both the MP20 and Tn51 strains were crushed, the contents were plated onto YM plates, and total colony-forming units (CFUs) were counted. In accordance with the microscopic observations, the total CFU from the Tn51 nodules was significantly less than that of the MP20 nodules (Table S1). These observations suggest that Tn51 can initiate nodule organogenesis later than MP20, but the nodules are not fully developed, and the bacteria do not form a functional symbiosis.

### A putative glycosyl transferase gene is required for the nodulation of *M. pudica* by MP20

**Fig 6:**
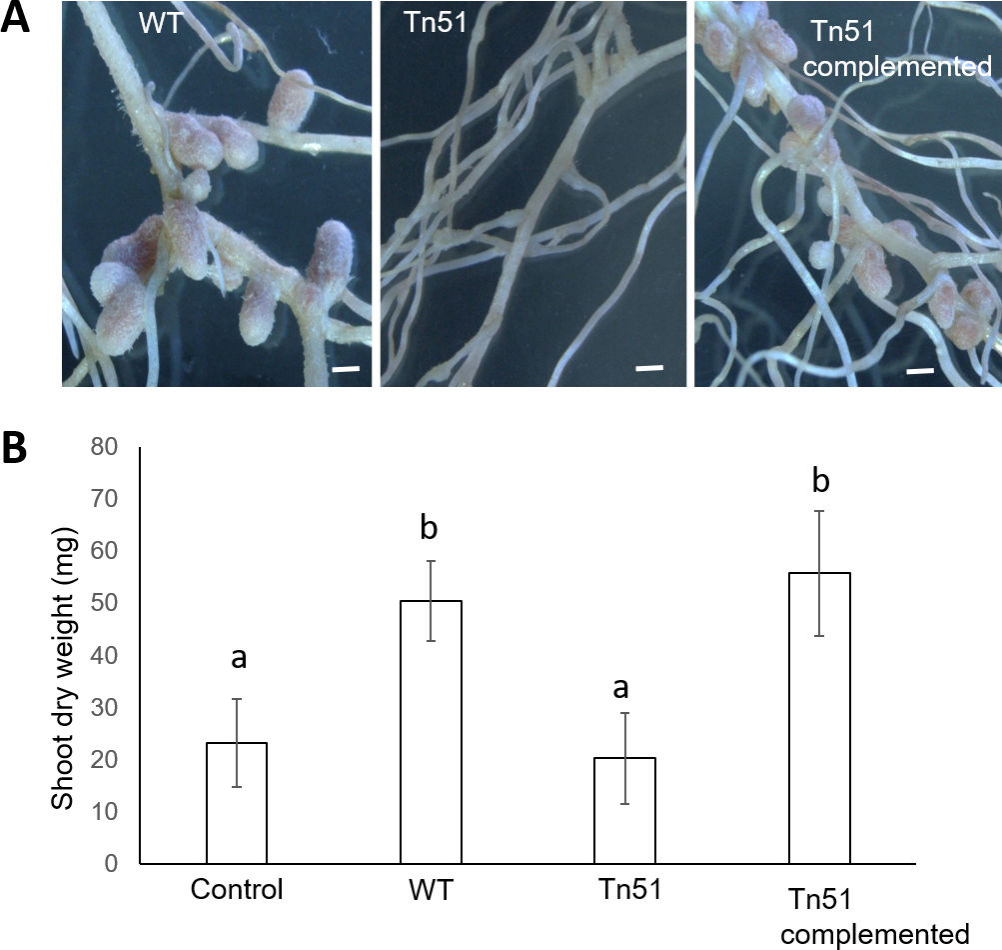
Complementation of Tn51 mutant with putative polysaccharide operon restores symbiosis. (A) Nodulation of *M. pudica* by MP20, Tn51, and Tn51 containing the wild-type operon (complemented). Note that the complemented Tn51 mutant strain forms nodules similar to WT. (B) Effect of inoculation with different strains on the growth of *M. pudica* in N-free condition. Increased growth reflects symbiotic N-fixation. Results are the mean ± standard deviation of three independent observations. Bars with different letters are significantly different (*P* < 0.01).

To identify the transposon-insertion site in the mutant, the genomic DNA flanking the transposon was cloned and sequenced, and the sequence was compared against the genome of *P. phymatum* STM815^T^ (26). The sequence analysis found that the transposon had disrupted BPHY_RS11650, annotated as a glycosyltransferase family 2 protein. BPHY_RS11650 is part of a cluster of four genes annotated as SDR family oxidoreductase, glycosyltransferase family 4 protein, and nucleoside-diphosphate sugar epimerase/dehydratase (Fig. S3). The genome organization of these genes suggests a multi-cistronic operon, and that transposon insertion in BPHY_RS11650 could lead to a polar effect on downstream genes. To determine if the nodulation defect is due to disruption in BPHY_RS11650, the Tn51 strain was complemented with the putative operon (BPHY_RS11650–BPHY_RS11635) cloned into pBBRMCS, inoculated onto *M. pudica,* and examined for nodulation and plant growth. The complemented strain formed nodules similar to MP20 (Fig. 6A), showing that the nodulation defect of Tn51 is due to insertion in BPHY_RS11650. The plants inoculated with the complemented Tn51 strain were symbiotically effective, as shown by the increase in shoot dry weight similar to the plants with MP20 (Fig. 6B).

### Rhamnose containing LPS O-antigen is involved in symbiotic interactions

The BPHY_RS11650 and BPHY_RS11635 genes are not assigned to any functional group in the KEGG database. However, further analysis of conserved protein domains in each of these genes revealed that the SDR family oxidoreductase (BPHY_RS11645) contains a WcaG domain that is conserved in proteins involved in cell envelope synthesis (45). The glycosyltransferase family 4 protein contained a GT_Wbl_Wbco_Like domain. Proteins with such a domain are involved in the synthesis of the O-antigen of LPS in *Pseudomonas aeruginosa* (46) and *Yersinia enterocolitica* (47). The nucleoside-diphosphate sugar epimerase/dehydratase shows similarity to *Staphylococcus aureus* CapD involved in the synthesis of capsular polysaccharide (48) and to the gene involved in LPS O-antigen synthesis in *Vibrio cholerae* (49).

**Fig 7:**
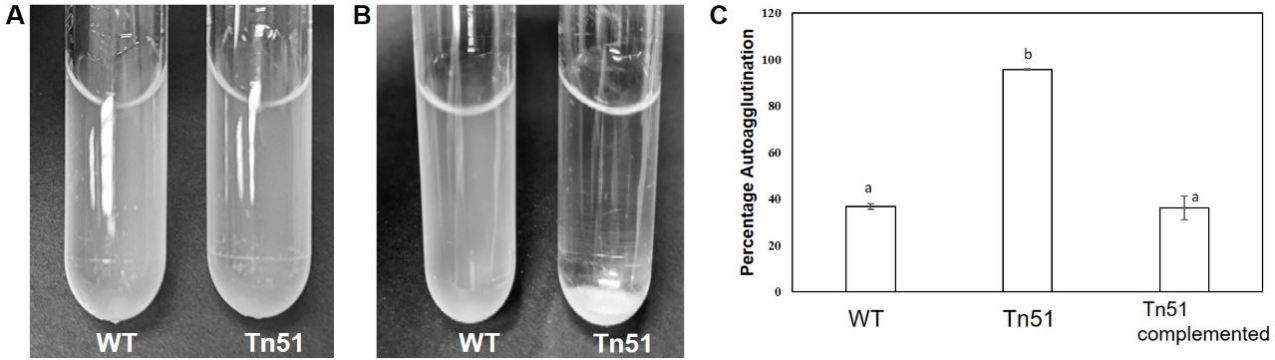
Agglutination of MP20, Tn51, and Tn51 complemented with putative polysaccharide operon. (A) Cells grown in TY and (B) cells grown in TY containing 1% NaCl. Note that the Tn51 mutant agglutinates at the bottom only in media containing NaCl. (C) Agglutination was reduced in the Tn51 mutant complemented with the wild-type polysaccharide operon.

This analysis indicated that Tn51 could likely have a defect in cell envelope components such as the O-antigen of LPS. In alpha rhizobia, LPS is an important determinant of nodulation and symbiotic N-fixation (50–52). To determine if Tn51 is affected in LPS synthesis, an auto- agglutination test was used. At high salt concentrations, defects in LPS lead to cell agglutination due to the inability of the bacteria to maintain hydrophobicity (53). The MP20 and Tn51 strains were grown in TY, then transferred to TY containing 1% NaCl, and incubated statically. No agglutination was observed in cells growing in TY (Fig. 7A), but in the presence of 1% salt, the Tn51 showed increased agglutination, and the cells settled at the bottom of the wells, but this did not occur with the MP20 strain (Fig. 7B). The agglutination was alleviated by complementing the Tn51 strain with the wild-type operon (Fig. 7C). Taken together, this suggests that Tn51 is likely affected in LPS synthesis.

**Fig 8:**
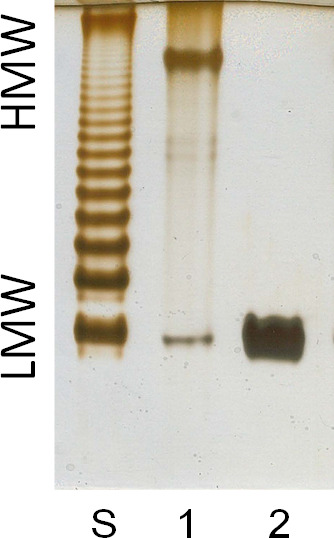
LPS analysis of MP20 and Tn51 mutant. (A) Silver staining showing the presence of high molecular weight (HMW) and low molecular weight (LMW) bands in the LPS from MP20 (WT) and lack of HMW and increase in LMW in the Tn51 mutant extracted from cells (compare lanes 1 and 2). S: LPS from *Salmonella* Typhimurium.

To further demonstrate LPS defect, we extracted and analyzed LPS from MP20 and Tn51 using SDS-polyacrylamide gel electrophoresis and silver staining. The recovery of LPS extracted from the Tn51 mutant was significantly lower (a yield percentage of LPS was 1.85% for MP20 and 0.5% for Tn51). In accordance with the low yield, the LPS of TN51 showed a significant change in the electrophoretic profile compared to that of the MP20 strain (Fig. 8A, lane 1). LPS from MP20 formed a single high molecular weight (HMW) band, a faint ladder in the mid-section of the gel, and a narrow low molecular weight (LMW) band at the bottom. In contrast, LPS from Tn51 showed only a high-intensity LMW band (Fig. 8A, lane 2). The absence of the HMW bands in Tn51 suggests a significant change in the structure of LPS, suggesting loss of the O-antigen.

To better understand the changes in the LPS profiles in the PAGE and to correlate these modifications with the observed biological phenotypes, we looked at the glycosyl and fatty acid composition of the purified LPS. The major glycosyl residue found in the MP20 LPS was rhamnose, suggesting it could be an HMW O-antigen polymer constituent. In addition, the LPS consisted of glucose, heptose, 4-aminoarabinose, ribose, and trace amounts of galactose and N-acetyl-quinovosamine (molar ratios listed in Table S2). We also detected other neutral sugar residues that we could not fully assign due to the lack of the corresponding original standards. In contrast to MP20, the LPS from Tn51 was depleted of rhamnose and showed a significant increase in the content of glucose, galactose, heptose, 4-aminoarabinose, and N-acetyl-glucosamine (Table S2). The last two sugars are likely associated with lipid A. Similar to the MP20 LPS, the content of N-acetyl-quinovosamine was also low (Table S2). Both LPS demonstrated similar relative ratios of 3-hydroxy-tetradecanoic acid [14:0(3-OH)], 3-hydroxy hexadecanoic acid [16:0(3-OH)], and palmitic acid [16:0], suggesting no significant changes to the lipid A portion of LPS.

The lack of rhamnose in the O-antigen of Tn51 LPS and the significant increase in the relative ratio of other glycosyl residues (likely associated with the lipid A and core region of LPS) together with the observed lack of HMW band LPS and increase in the relative intensity of LMW bands in PAGE of Tn51 (Fig. 8, lanes 1 and 2) strongly indicate that the Tn51 mutant is deprived of rhamnose enriched O-antigen. More structural work is needed to fully define the structure of the O-chain, core, and lipid A of *P. phymatum*.

## DISCUSSION

*P. phymatum* is a β-proteobacteria that has a relatively broad host range for forming N-fixing symbiotic associations with legumes, especially *Mimosa* species, but also with several other mimosoids and even some papilionoids (12, 14, 25, 26). However, the mechanisms important for symbiosis are just beginning to be determined. Earlier studies used transposon mutagenesis and showed that genes involved in glycolysis and synthesis of branched -chain amino acids are important for the nodulation of *M. pudica* by *P. phymatum* and *C. taiwanensis* (54, 55). In this study, we determined the genome of MP20, a strain isolated from the nodules of field -grown *M. pudica* (24). Genome analysis showed that MP20 was 99.9% identical to the *P. phymatum* STM815^T^ that was initially isolated from the nodules of papilionoid legume *Machaerium Lunatum*. We then generated transposon insertion mutants of MP20 and screened them for defects in nodulation. To reduce the number of mutants for screening, the mutants were isolated on minimal media, thus eliminating all auxotrophs. After multiple screenings, we identified a mutant (Tn51) that showed delayed and ineffective nodulation. Light and electron microscopy showed that the nodules formed by the mutant were deformed and were likely compromised in their symbiotic compatibility with *M. pudica*.

The transposon insertion was localized to a gene encoding glycosyltransferase family 2 protein that is not assigned to a functional group. The disrupted gene was within a cluster of genes annotated as glycosyltransferases that could be involved in synthesizing polysaccharides and/or LPS. In accordance with a defect in LPS, the Tn51 mutant showed increased agglutination in the presence of high salt. LPS, a major component of the outer membrane of Gram-negative bacteria, contains a conserved core and a variable O-antigen (56, 57).

Analysis of transcriptomic changes of rhizobia in the rhizospshere of *M. pudica* suggested that LPS is likely to be a factor determining the competitive colonization of roots (58). In support of this hypothesis, this study shows that the O-antigen of MP20 is involved in symbiotic interaction with *M. pudica*. We also show that the O-antigen of MP20 contains high amounts of rhamnose. Similarly, the O-antigen of the related *Burkholderia cepacia* was shown to contain rhamnose (59). However, we did not detect a gene in the *Cupriavidus taiwanensis* LMG 19424 genome with significant similarity to the gene disrupted by the transposon in the Tn51 strain. This suggests that this related β-rhizobium, which also nodulates *M. pudica*, might utilize different genes for synthesizing O-antigen. The putative pathway of the O-antigen nucleotide sugars in *C. taiwanensis* involves the synthesis of dDTP Rhamnose using *rmlD*.

Earlier studies have demonstrated that the O-antigen is important for nodulation by various alpha-rhizobia (38, 60–63), and this study demonstrates that O-antigen is also important for nodulation by beta-rhizobia. Our analysis shows that the LPS of *P. phymatum* MP20 contains rhamnose-rich O-antigen that is important for the development of nodules and further multiplication and persistence of bacteria within the nodules. These results are similar to earlier studies showing the requirement of O-antigen for nodulation of *Sesbania cannabina* by *Rhizobium* sp. IRBG74 (38) and *S. rostrata* by *Azorhizobium caulinodans* ORS571 (64, 65).

As LPS is a structural component of the outer membrane of Gram-negative bacteria, the alteration in O-antigen could affect bacterial survival in stress conditions, especially those that affect the cell envelope (66). However, evidence has been presented that rhizobial LPS is likely involved in signaling and communication with the legume host (64). LPS was shown to be involved in early communication and binding to the roots by *R. leguminosarum* (67). Noel et al. (61) postulated that LPS is recognized by a plant membrane receptor in a structure-dependent manner that is required for further infection. In addition, the synthesis of the rhamnose-rich O-antigen of *Sinorhizobium fredii* NGR234 is induced by flavonoids and is required for symbiosis with different host plants (68). We have earlier shown that a mutant of *Rhizobium* sp. IRBG74, lacking rhamnose in its O-antigen, formed small and partially effective nodules on *S. cannabina* that contained prematurely senescing bacteroids (38). In accordance with these earlier studies and based on the results of this study, we postulate that the O-antigen of *P. phymatum* is likely to be important for effective communication during infection and nodulation of *M. pudica*. Further research is needed to determine if the lack of O-antigen leads to defects in signal exchange and/or bacteroid differentiation and survival.

## SUPPLEMENTAL MATERIAL

Figure S1 jb.00422-24-s0001.png Tree inferred with FastME 2.1.6.1 from GBDP distances calculated from genome sequences.

Figure S2 jb.00422-24-s0002.png Average Nucleotide Identity (ANI) using both best hits (one-way ANI) and reciprocal best hits (two-way ANI).

Figure S3 jb.00422-24-s0003.jpg Genomic organization of a putative operon disrupted by the Himar transposon in the TN51 mutant of MP20.

Tables S1 and S2 jb.00422-24-s0004.docx CFU from nodules and mol% of sugars in LPS.

Legends jb.00422-24-s0005.docx Legends for supplemental tables and figures.

ASM does not own the copyrights to Supplemental Material that may be linked to, or accessed through, an article. The authors have granted ASM a non-exclusive, world-wide license to publish the Supplemental Material files. Please contact the corresponding author directly for reuse.

## References

[1] Graham PH, Vance CP. 2003. Legumes: importance and constraints to greater use. Plant Physiol 131:872–877. doi:10.1104/pp.017004 12644639 PMC1540286

[2] Peoples MB, Brockwell J, Herridge DF, Rochester IJ, Alves BJR, Urquiaga S, Boddey RM, Dakora FD, Bhattarai S, Maskey SL, Sampet C, Rerkasem B, Khan DF, Hauggaard-Nielsen H, Jensen ES. 2009. The contributions of nitrogen-fixing crop legumes to the productivity of agricultural systems. Symbiosis 48:1–17. doi:10.1007/BF03179980

[3] Gyaneshwar P, Hirsch AM, Moulin L, Chen W-M, Elliott GN, Bontemps C, Estrada-de Los Santos P, Gross E, Dos Reis FB, Sprent JI, Young JPW, James EK. 2011. Legume-nodulating betaproteobacteria: diversity, host range, and future prospects. Mol Plant Microbe Interact 24:1276–1288. doi:10.1094/MPMI-06-11-0172 21830951

[4] Oldroyd GED. 2013. Speak, friend, and enter: signalling systems that promote beneficial symbiotic associations in plants. Nat Rev Microbiol 11:252–263. doi:10.1038/nrmicro2990 23493145

[5] Venkateshwaran M, Volkening JD, Sussman MR, Ané JM. 2013. Symbiosis and the social network of higher plants. Curr Opin Plant Biol 16:118–127. doi:10.1016/j.pbi.2012.11.007 23246268

[6] Dénarié J, Debellé F, Rosenberg C. 1992. Signaling and host range variation in nodulation. Annu Rev Microbiol 46:497–531. doi:10.1146/annurev.mi.46.100192.002433 1444265

[7] Madsen JS, Sørensen SJ, Burmølle M. 2018. Bacterial social interactions and the emergence of community-intrinsic properties. Curr Opin Microbiol 42:104–109. doi:10.1016/j.mib.2017.11.018 29197823

[8] Madsen EB, Madsen LH, Radutoiu S, Olbryt M, Rakwalska M, Szczyglowski K, Sato S, Kaneko T, Tabata S, Sandal N, Stougaard J. 2003. A receptor kinase gene of the LysM type is involved in legumeperception of rhizobial signals. Nat New Biol 425:637–640. doi:10.1038/nature02045 14534591

[9] Sawada H, Kuykendall LD, Young JM. 2003. Changing concepts in the systematics of bacterial nitrogen-fixing legume symbionts. J Gen Appl Microbiol 49:155–179. doi:10.2323/jgam.49.155 12949698

[10] Velázquez E, García-Fraile P, Ramírez-Bahena M-H, Rivas R, Martínez-Molina E. 2010. Bacteria involved in nitrogen-fixing legume symbiosis: current taxonomic perspective, p 1–25. In Khan MS, Musarrat J, Zaidi A (ed), Microbes for legume improvement. Springer Vienna, Vienna.

[11] Chen WM, James EK, Prescott AR, Kierans M, Sprent JI. 2003. Nodulation of Mimosa spp. by the β-Proteobacterium Ralstonia taiwanensis. Mol Plant Microbe Interact 16:1051–1061. doi:10.1094/MPMI.2003.16.12.1051 14651338

[12] Elliott GN, Chen W-M, Chou J-H, Wang H-C, Sheu S-Y, Perin L, Reis VM, Moulin L, Simon MF, Bontemps C, Sutherland JM, Bessi R, de Faria SM, Trinick MJ, Prescott AR, Sprent JI, James EK. 2007. Burkholderia phymatum is a highly effective nitrogen-fixing symbiont of Mimosa spp. and fixes nitrogen ex planta. New Phytol 173:168–180. doi:10.1111/j.1469-8137.2006.01894.x 17176403

[13] Bontemps C, Elliott GN, Simon MF, Dos Reis Júnior FB, Gross E, Lawton RC, Neto NE, de Fátima Loureiro M, De Faria SM, Sprent JI, James EK, Young JPW. 2010. Burkholderia species are ancient symbionts of legumes. Mol Ecol 19:44–52. doi:10.1111/j.1365-294X.2009.04458.x 20002602

[14] dos Reis Jr FB, Simon MF, Gross E, Boddey RM, Elliott GN, Neto NE, de Fatima Loureiro M, de Queiroz LP, Scotti MR, Chen W, Norén A, Rubio MC, de Faria SM, Bontemps C, Goi SR, Young JPW, Sprent JI, James EK. 2010. Nodulation and nitrogen fixation by Mimosa spp. in the Cerrado and Caatinga biomes of Brazil. New Phytol 186:934–946. doi:10.1111/j.1469-8137.2010.03267.x 20456044

[15] Bournaud C, de Faria SM, dos Santos JMF, Tisseyre P, Silva M, Chaintreuil C, Gross E, James EK, Prin Y, Moulin L. 2013. Burkholderia species are the most common and preferred nodulating symbionts of the Piptadenia group (Tribe Mimoseae). PLoS ONE 8:e63478. doi:10.1371/journal.pone.0063478 23691052 PMC3655174

[16] Elliott GN, Chen WM, Bontemps C, Chou JH, Young JPW, Sprent JI, James EK. 2007. Nodulation of Cyclopia spp. (Leguminosae, Papilionoideae) by Burkholderia tuberum. Ann Bot 100:1403–1411. doi:10.1093/aob/mcm227 17881339 PMC2759218

[17] Garau G, Yates RJ, Deiana P, Howieson JG. 2009. Novel strains of nodulating Burkholderia have a role in nitrogen fixation with papilionoid herbaceous legumes adapted to acid, infertile soils. Soil Biol Biochem 41:125–134. doi:10.1016/j.soilbio.2008.10.011

[18] Beukes CW, Venter SN, Law IJ, Phalane FL, Steenkamp ET. 2013. South African papilionoid legumes are nodulated by diverse Burkholderia with unique nodulation and nitrogen-fixation loci. PLoS ONE 8:e68406. doi:10.1371/journal.pone.0068406 23874611 PMC3708930

[19] Lemaire B, Van Cauwenberghe J, Chimphango S, Stirton C, Honnay O, Smets E, Muasya AM. 2015. Recombination and horizontal transfer of nodulation and ACC deaminase (acdS) genes within Alpha- and Betaproteobacteria nodulating legumes of the Cape Fynbos biome. FEMS Microbiol Ecol 91:1–11. doi:10.1093/femsec/fiv118 26433010

[20] Lemaire B, Chimphango SBM, Stirton C, Rafudeen S, Honnay O, Smets E, Chen W-M, Sprent J, James EK, Muasya AM. 2016. Biogeographical patterns of Legume-nodulating Burkholderia spp.: from African Fynbos to continental scales. Appl Environ Microbiol 82:5099–5115. doi:10.1128/AEM.00591-16 27316955 PMC4988186

[21] Lardi M, de Campos SB, Purtschert G, Eberl L, Pessi G. 2017. Competition experiments for legume infection identify Burkholderia phymatum as a highly competitive β-Rhizobium. Front Microbiol 8:1527. doi:10.3389/fmicb.2017.01527 28861050 PMC5559654

[22] Talbi C, Delgado MJ, Girard L, Ramírez-Trujillo A, Caballero-Mellado J, Bedmar EJ. 2010. Burkholderia phymatum strains capable of nodulating Phaseolus vulgaris are present in moroccan soils. Appl Environ Microbiol 76:4587–4591. doi:10.1128/AEM.02886-09 20472732 PMC2897466

[23] Elliott GN, Chou J-H, Chen W-M, Bloemberg GV, Bontemps C, Martínez-Romero E, Velázquez E, Young JPW, Sprent JI, James EK. 2009. Burkholderia spp. are the most competitive symbionts of Mimosa, particularly under N-limited conditions. Environ Microbiol 11:762–778. doi:10.1111/j.1462-2920.2008.01799.x 19040456

[24] Gehlot HS, Tak N, Kaushik M, Mitra S, Chen W-M, Poweleit N, Panwar D, Poonar N, Parihar R, Tak A, Sankhla IS, Ojha A, Rao SR, Simon MF, Reis Junior FBD, Perigolo N, Tripathi AK, Sprent JI, Young JPW, James EK, Gyaneshwar P. 2013. An invasive Mimosa in India does not adopt the symbionts of its native relatives. Ann Bot 112:179–196. doi:10.1093/aob/mct112 23712450 PMC3690997

[25] Bellés-Sancho P, Beukes C, James EK, Pessi G. 2023. Nitrogen-fixing symbiotic Paraburkholderia species: current knowledge and future perspectives. N 4:135–158. doi:10.3390/nitrogen4010010

[26] Moulin L, Klonowska A, Caroline B, Booth K, Vriezen JAC, Melkonian R, James EK, Young JPW, Bena G, Hauser L, Land M, Kyrpides N, Bruce D, Chain P, Copeland A, Pitluck S, Woyke T, Lizotte-Waniewski M, Bristow J, Riley M. 2014. Complete genome sequence of Burkholderia phymatum STM815T, a broad host range and efficient nitrogen-fixing symbiont of Mimosa species. Stand Genomic Sci 9:763–774. doi:10.4056/sigs.4861021 25197461 PMC4148976

[27] Coutinho BG, Mitter B, Talbi C, Sessitsch A, Bedmar EJ, Halliday N, James EK, Cámara M, Venturi V. 2013. Regulon studies and in planta role of the BraI/R quorum-sensing system in the plant-beneficial Burkholderia cluster. Appl Environ Microbiol 79:4421–4432. doi:10.1128/AEM.00635-13 23686262 PMC3697526

[28] de Campos SB, Lardi M, Gandolfi A, Eberl L, Pessi G. 2017. Mutations in two Paraburkholderia phymatum type VI secretion systems cause reduced fitness in interbacterial competition. Front Microbiol 8:2473. doi:10.3389/fmicb.2017.02473 29312183 PMC5732942

[29] Lardi M, Aguilar C, Pedrioli A, Omasits U, Suppiger A, Cárcamo-Oyarce G, Schmid N, Ahrens CH, Eberl L, Pessi G. 2015. σ^54^-Dependent response to nitrogen limitation and virulence in Burkholderia cenocepacia strain H111. Appl Environ Microbiol 81:4077–4089. doi:10.1128/AEM.00694-15 25841012 PMC4524130

[30] Glazebrook J, Walker GC. 1991. Genetic techniques in Rhizobium meliloti. Methods Enzymol 204:398–418. doi:10.1016/0076-6879(91)04021-f 1658566

[31] Yoon S-H, Ha S-M, Lim J, Kwon S, Chun J. 2017. A large-scale evaluation of algorithms to calculate average nucleotide identity. Antonie Van Leeuwenhoek 110:1281–1286. doi:10.1007/s10482-017-0844-4 28204908

[32] Richter M, Rosselló-Móra R, Oliver Glöckner F, Peplies J. 2016. JSpeciesWS: a web server for prokaryotic species circumscription based on pairwise genome comparison. Bioinformatics 32:929–931. doi:10.1093/bioinformatics/btv681 26576653 PMC5939971

[33] Jain C, Dilthey A, Koren S, Aluru S, Phillippy AM. 2018. A fast approximate algorithm for mapping long reads to large reference databases. J Comput Biol 25:766–779. doi:10.1089/cmb.2018.0036 29708767 PMC6067103

[34] Lefort V, Desper R, Gascuel O. 2015. FastME 2.0: a comprehensive, accurate, and fast distance-based phylogeny inference program. Mol Biol Evol 32:2798–2800. doi:10.1093/molbev/msv150 26130081 PMC4576710

[35] Meier-Kolthoff JP, Göker M. 2019. TYGS is an automated high-throughput platform for state-of-the-art genome-based taxonomy. Nat Commun 10:2182. doi:10.1038/s41467-019-10210-3 31097708 PMC6522516

[36] Meier-Kolthoff JP, Auch AF, Klenk HP, Göker M. 2013. Genome sequence-based species delimitation with confidence intervals and improved distance functions. BMC Bioinformatics 14:60. doi:10.1186/1471-2105-14-60 23432962 PMC3665452

[37] Perry BJ, Yost CK. 2014. Construction of a mariner-based transposon vector for use in insertion sequence mutagenesis in selected members of the Rhizobiaceae. BMC Microbiol 14:298. doi:10.1186/s12866-014-0298-z 25433486 PMC4255674

[38] Mitra S, Mukherjee A, Wiley-Kalil A, Das S, Owen H, Reddy PM, Ané JM, James EK, Gyaneshwar P. 2016. A rhamnose-deficient lipopolysaccharide mutant of Rhizobium sp. IRBG74 is defective in root colonization and beneficial interactions with its flooding-tolerant hosts Sesbania cannabina and wetland rice. J Exp Bot 67:5869–5884. doi:10.1093/jxb/erw354 27702995

[39] Cummings SP, Gyaneshwar P, Vinuesa P, Farruggia FT, Andrews M, Humphry D, Elliott GN, Nelson A, Orr C, Pettitt D, Shah GR, Santos SR, Krishnan HB, Odee D, Moreira FMS, Sprent JI, Young JPW, James EK. 2009. Nodulation of Sesbania species by Rhizobium (Agrobacterium) strain IRBG74 and other rhizobia. Environ Microbiol 11:2510–2525. doi:10.1111/j.1462-2920.2009.01975.x 19555380 PMC7163632

[40] Westphal O, Jann K. 1965. Bacterial lipopolysaccharides. Extraction with phenol-water and further applications of the procedure. Methods Carbohydr Chem 5:83–91.

[41] Krauss JH, Weckesser J, Mayer H. 1988. Electrophoretic analysis of lipopolysaccharides of purple nonsulfur bacteria. Int J Syst Bacteriol 38:157–163. doi:10.1099/00207713-38-2-157

[42] Corzo J, Pérez-Galdona R, León-Barrios M, Gutiérrez-Navarro AM. 1991. Alcian blue fixation allows silver staining of the isolated polysaccharide component of bacterial lipopolysaccharides in polyacrylamide gels. Electrophoresis 12:439–441. doi:10.1002/elps.1150120611 1716199

[43] Muszynski A, Laus M, Kijne JW, Carlson RW. 2011. Structures of the lipopolysaccharides from Rhizobium leguminosarum RBL5523 and its UDP-glucose dehydrogenase mutant (exo5). Glycobiology 21:55–68. doi:10.1093/glycob/cwq131 20817634 PMC2998983

[44] Bhat UR, Mayer H, Yokota A, Hollingsworth RI, Carlson RW. 1991. Occurrence of lipid A variants with 27-hydroxyoctacosanoic acid in lipopolysaccharides from members of the family Rhizobiaceae. J Bacteriol 173:2155–2159. doi:10.1128/jb.173.7.2155-2159.1991 2007543 PMC207761

[45] Wang J, Chitsaz F, Derbyshire MK, Gonzales NR, Gwadz M, Lu S, Marchler GH, Song JS, Thanki N, Yamashita RA, Yang M, Zhang D, Zheng C, Lanczycki CJ, Marchler-Bauer A. 2023. The conserved domain database in 2023. Nucleic Acids Res 51:D384–D388. doi:10.1093/nar/gkac1096 36477806 PMC9825596

[46] Burrows LL, Charter DF, Lam JS. 1996. Molecular characterization of the Pseudomonas aeruginosa serotype O5 (PAO1) B-band lipopolysaccharide gene cluster. Mol Microbiol 22:481–495. doi:10.1046/j.1365-2958.1996.1351503.x 8939432

[47] Skurnik M, Venho R, Toivanen P, al-Hendy A. 1995. A novel locus of Yersinia enterocolitica serotype O:3 involved in lipopolysaccharide outer core biosynthesis. Mol Microbiol 17:575–594. doi:10.1111/j.1365-2958.1995.mmi_17030575.x 8559076

[48] O’Riordan K, Lee JC. 2004. Staphylococcus aureus capsular polysaccharides. Clin Microbiol Rev 17:218–234. doi:10.1128/CMR.17.1.218-234.2004 14726462 PMC321462

[49] Fallarino A, Mavrangelos C, Stroeher UH, Manning PA. 1997. Identification of additional genes required for O-antigen biosynthesis in Vibrio cholerae O1. J Bacteriol 179:2147–2153. doi:10.1128/jb.179.7.2147-2153.1997 9079898 PMC178949

[50] Price NPJ. 1999. Carbohydrate determinants of Rhizobium-legume symbioses. Carbohydr Res 317:1–9. doi:10.1016/s0008-6215(99)00075-0 10466203

[51] Noel KD, Duelli DM. 2000. *Rhizobium* lipopolysaccharide and its role in symbiosis, p 415–431. In Prokaryotic nitrogen fixation: a model system for the analysis of a biological process. Horizon Scientific Press, Wymondham.

[52] Fraysse N, Couderc F, Poinsot V. 2003. Surface polysaccharide involvement in establishing the Rhizobium-legume symbiosis. Eur J Biochem 270:1365–1380. doi:10.1046/j.1432-1033.2003.03492.x 12653992

[53] Azimi S, Thomas J, Cleland SE, Curtis JE, Goldberg JB, Diggle SP. 2021. O-specific antigen-dependent surface hydrophobicity mediates aggregate assembly type in Pseudomonas aeruginosa. MBio 12:e0086021. doi:10.1128/mBio.00860-21 34372703 PMC8406328

[54] Chen WM, Prell J, James EK, Sheu DS, Sheu SY. 2012. Biosynthesis of branched-chain amino acids is essential for effective symbioses between betarhizobia and Mimosa pudica. Microbiol (Reading) 158:1758–1766. doi:10.1099/mic.0.058370-0 22556357

[55] Chen WM, Prell J, James EK, Sheu DS, Sheu SY. 2012. Effect of phosphoglycerate mutase and fructose 1,6-bisphosphatase deficiency on symbiotic Burkholderia phymatum. Microbiol (Reading) 158:1127–1136. doi:10.1099/mic.0.055095-0 22282515

[56] Raetz CRH, Whitfield C. 2002. Lipopolysaccharide endotoxins. Annu Rev Biochem 71:635–700. doi:10.1146/annurev.biochem.71.110601.135414 12045108 PMC2569852

[57] Carlson RW, Forsberg LS, Kannenberg EL. 2010. Lipopolysaccharides in *Rhizobium*-legume symbioses, p 339–386. In Wang X, Quinn PJ (ed), Endotoxins: structure, function and recognition. Springer International Publishing. 10.1007/978-90-481-9078-2_16 20593275

[58] Klonowska A, Melkonian R, Miché L, Tisseyre P, Moulin L. 2018. Transcriptomic profiling of Burkholderia phymatum STM815, Cupriavidus taiwanensis LMG19424 and Rhizobium mesoamericanum STM3625 in response to Mimosa pudica root exudates illuminates the molecular basis of their nodulation competitiveness and symbiotic evolutionary history. BMC Genomics 19:105. doi:10.1186/s12864-018-4487-2 29378510 PMC5789663

[59] Beynon LM, Perry MB. 1993. Structure of the lipopolysaccharide O-antigen of Pseudomonas cepacia serotype A. Biochem Cell Biol 71:417–420. doi:10.1139/o93-061 7514883

[60] Becker A, Fraysse N, Sharypova L. 2005. Recent advances in studies on structure and symbiosis-related function of rhizobial K-antigens and lipopolysaccharides. Mol Plant Microbe Interact 18:899–905. doi:10.1094/MPMI-18-0899 16167760

[61] Noel KD, Box JM, Bonne VJ. 2004. 2-O-methylation of fucosyl residues of a rhizobial lipopolysaccharide is increased in response to host exudate and is eliminated in a symbiotically defective mutant. Appl Environ Microbiol 70:1537–1544. doi:10.1128/AEM.70.3.1537-1544.2004 15006776 PMC368319

[62] Ojeda KJ, Box JM, Noel KD. 2010. Genetic basis for Rhizobium etli CE3 O-antigen O-methylated residues that vary according to growth conditions. J Bacteriol 192:679–690. doi:10.1128/JB.01154-09 19948805 PMC2812443

[63] Tang Z, Cai S, Liu Y, Li D, Xie F, Lin H, Chen D, Li Y. 2023. A lipopolysaccharide O-antigen synthesis gene in Mesorhizobium huakuii plays differentiated roles in root nodule symbiotic compatibility with Astragalus sinicus. Mol Plant Microbe Interact 36:623–635. doi:10.1094/MPMI-05-23-0066-R 37366577

[64] Gao M, D’Haeze W, De Rycke R, Wolucka B, Holsters M. 2001. Knockout of an azorhizobial dTDP-L-rhamnose synthase affects lipopolysaccharide and extracellular polysaccharide production and disables symbiosis with Sesbania rostrata. Mol Plant Microbe Interact 14:857–866. doi:10.1094/MPMI.2001.14.7.857 11437259

[65] Mathis R, Van Gijsegem F, De Rycke R, D’Haeze W, Van Maelsaeke E, Anthonio E, Van Montagu M, Holsters M, Vereecke D. 2005. Lipopolysaccharides as a communication signal for progression of legume endosymbiosis. Proc Natl Acad Sci U S A 102:2655–2660. doi:10.1073/pnas.0409816102 15699329 PMC549025

[66] Joiner KA. 1985. Studies on the mechanism of bacterial resistance to complement-mediated killing and on the mechanism of action of bactericidal antibody, p 99–133. In Loos M (ed), Bacteria and complement. Springer, Berlin Heidelberg, Berlin, Heidelberg. 10.1007/978-3-642-45604-6_6 3910367

[67] Dazzo FB, Brill WJ. 1979. Bacterial polysaccharide which binds Rhizobium trifolii to clover root hairs. J Bacteriol 137:1362–1373. doi:10.1128/jb.137.3.1362-1373.1979 86535 PMC218321

[68] Reuhs BL, Relić B, Forsberg LS, Marie C, Ojanen-Reuhs T, Stephens SB, Wong CH, Jabbouri S, Broughton WJ. 2005. Structural characterization of a flavonoid-inducible Pseudomonas aeruginosa A-band-like O antigen of Rhizobium sp. strain NGR234, required for the formation of nitrogen-fixing nodules. J Bacteriol 187:6479–6487. doi:10.1128/JB.187.18.6479-6487.2005 16159781 PMC1236632

