# Supplementary figures and images for: A rhamnose-rich O-antigen of Paraburkholderia phymatum MP20 is required for symbiosis with Mimosa pudica
Source: J Bacteriol. 2025 Jan 23;207(2):e00422-24. doi: 10.1128/jb.00422-24 (PMC11841133; doi:10.1128/jb.00422-24)

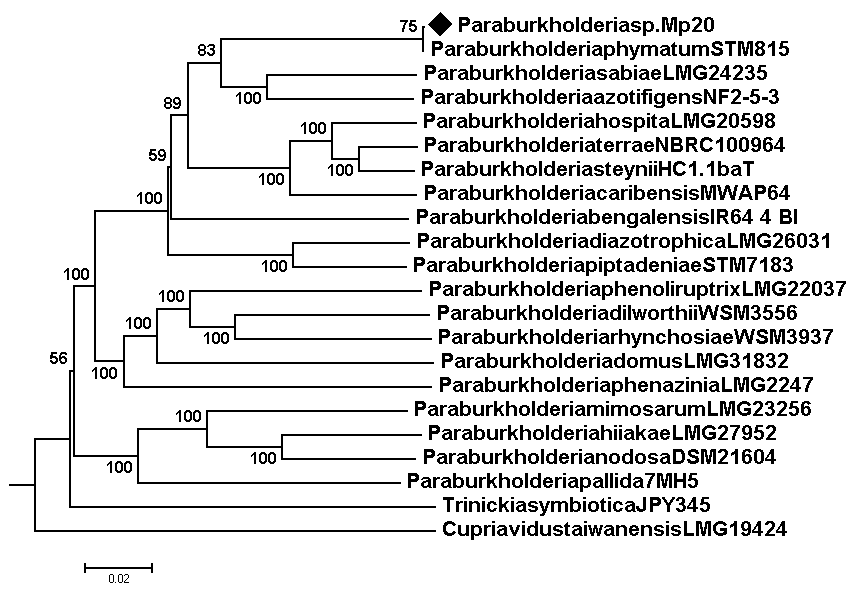

Supplement: Figure S1 — Tree inferred with FastME 2.1.6.1 from GBDP distances calculated from genome sequences. [file jb.00422-24-s0001.png]

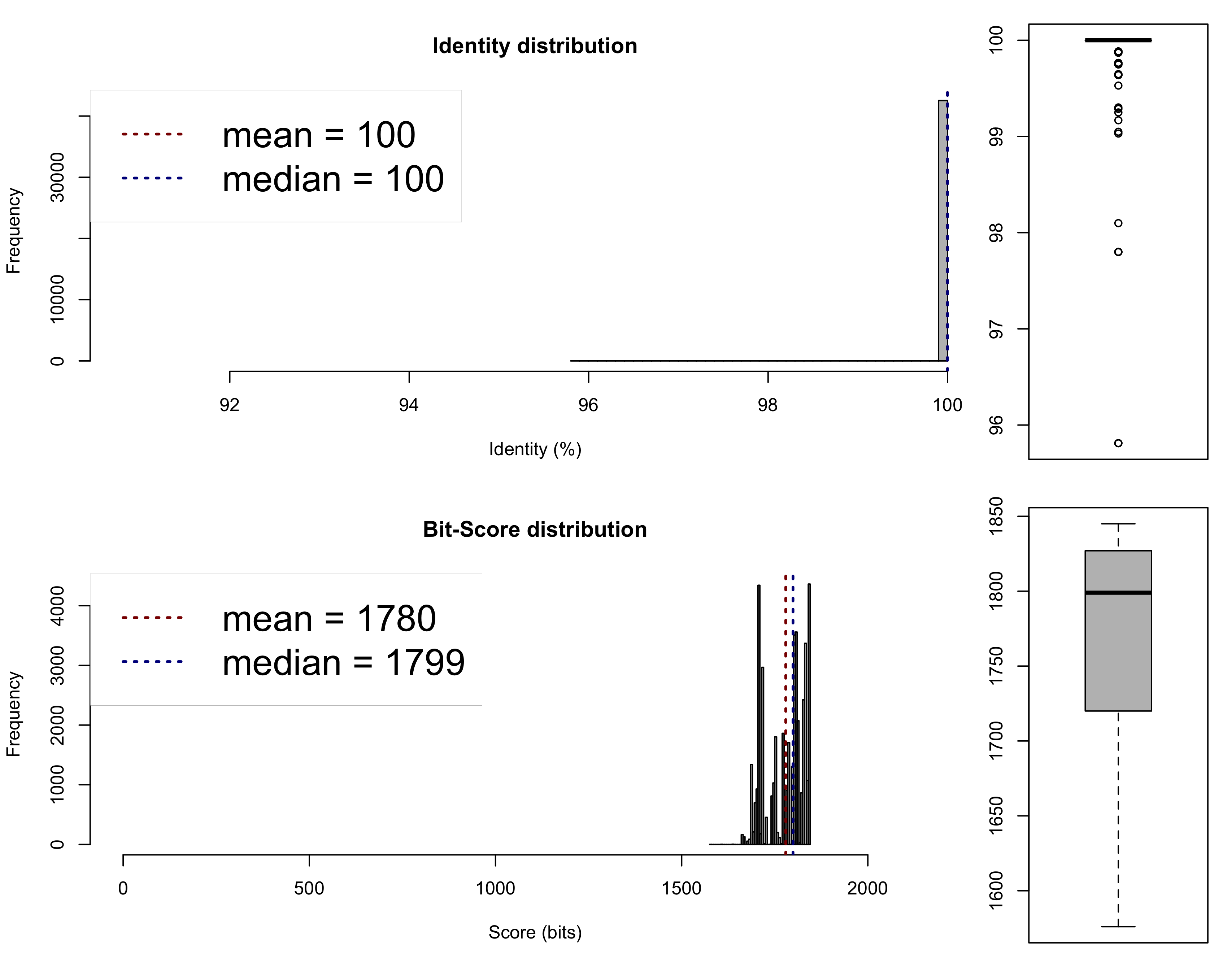

Supplement: Figure S2 — Average Nucleotide Identity (ANI) using both best hits (one-way ANI) and reciprocal best hits (two-way ANI). [file jb.00422-24-s0002.png]

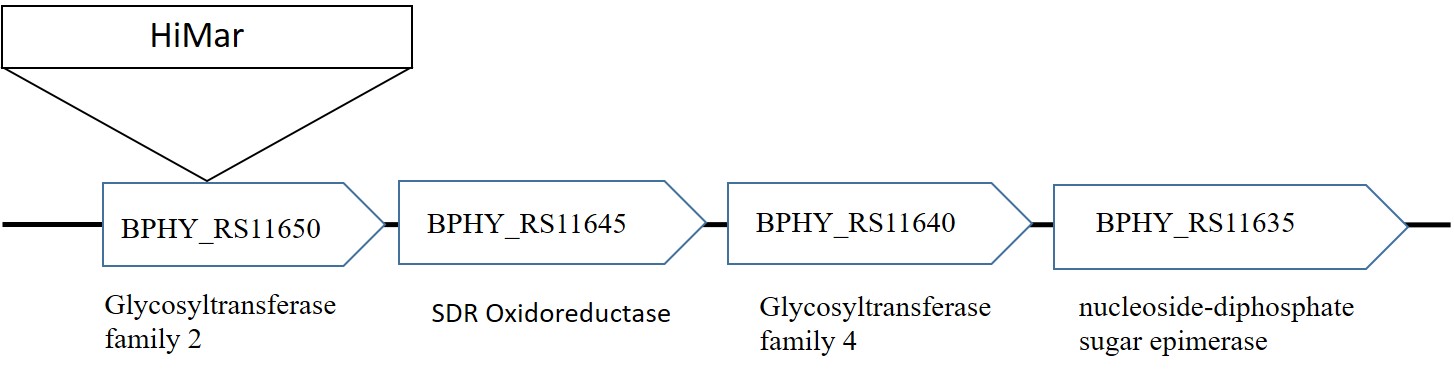

Supplement: Figure S3 — Genomic organization of a putative operon disrupted by the Himar transposon in the TN51 mutant of MP20. [file jb.00422-24-s0003.jpg]
