# Supplementary material for: A rhamnose-rich O-antigen of Paraburkholderia phymatum MP20 is required for symbiosis with Mimosa pudica
Source: J Bacteriol. 2025 Jan 23;207(2):e00422-24. doi: 10.1128/jb.00422-24 (PMC11841133; doi:10.1128/jb.00422-24)
Supplement: Tables S1 and S2 — CFU from nodules and mol% of sugars in LPS. [file jb.00422-24-s0004.docx]

| Strain | CFU/mg nodule |
| --- | --- |
| MP20 | 3.11 x 10^8^ |
| TN51 | 9.10 x 10^5^ |

**Table S1. Bacterial colonization of the *M. pudica* nodules**. 10 nodules from 3 different plants were pooled together, weighed, crushed and plated on YM plates. Total colony forming unites (CFU) were counted.

| Sugars | MP20 | Tn51 |
| --- | --- | --- |
|  |  |  |
| Ribose (Rib) | 2.8 | n.d. |
| Rhamnose (Rha) | 84.5 | n.d. |
| Mannose (Man) | n.d. | 0.3 |
| Glucose (Glc) | 3.4 | 36.4 |
| Galactose (Gal) | 0.3 | 5.0 |
| Heptose (Hep) | 7.8 | 37.9 |
| N-Acetylglucosamine (GlcNAc) | n.d. | 2.9 |
| 4-Aminoarabinose (Ara4N) | 1.2 | 17.4 |
| Sug 1 | + | n.d. |
| Sug 2 | + | n.d. |
| Sug 3 | + | n.d. |
| Sug 4 | + | + |

**Table S2**. **Sugar composition (Mole %) of LPS from MP20 and Tn51**
