# Supplementary material for: A rhamnose-rich O-antigen of Paraburkholderia phymatum MP20 is required for symbiosis with Mimosa pudica
Source: J Bacteriol. 2025 Jan 23;207(2):e00422-24. doi: 10.1128/jb.00422-24 (PMC11841133; doi:10.1128/jb.00422-24)
Supplement: Legends — for supplemental tables and figures. [file jb.00422-24-s0005.docx]

**Supplementary Data**

**Fig S1.** Tree inferred with FastME 2.1.6.1 from GBDP distances calculated from genome sequences. The branch lengths are scaled in terms of GBDP distance formula d5. The numbers above branches are GBDP pseudo-bootstrap support values > 60 % from 100 replications, with an average branch support of 92.7 %. The tree was rooted at the midpoint.

**FigS2.** Average Nucleotide Identity (ANI) using both best hits (one-way ANI) and reciprocal best hits (two-way ANI) between genomes of *Paraburkholderia* sp. MP 20 and *P. phymatum* STM 815^T^ using Kostas Lab ANI calculator.

**Fig S3.** Genomic organization of a putative operon disrupted by the Himar transposon in the TN51 mutant of MP20. The point of transposon insertion is shown.

**Table S1**. Bacterial cells recovered from the nodules formed by *P. phymatum* MP20 and Tn51 mutant.

**Table S2**. Sugar composition (mol%) of LPS extracted from MP20 and Tn51
